# FEV1/FEV6 Cutoff Points to Avoid False Negatives When Using Portable Devices, PICO-6^®^ and COPD-6^®^, in COPD Detection in Primary Healthcare Services

**DOI:** 10.3390/jcm14020576

**Published:** 2025-01-17

**Authors:** Miguel A. Hernandez-Mezquita, Idania De los Santos-Ventura, Vanesa Hidalgo-Sierra, Alfonso Pérez-Trullen, Ruth García Garcia, Tamara Clavero-Sánchez, Enrique Barrueco-Otero

**Affiliations:** 1 Pneumology Department, Salamanca University Hospital, 37007 Salamanca, Spain; idania7777@hotmail.com (I.D.l.S.-V.); rgarciag@saludcastillayleon.es (R.G.G.); tclaveros@saludcastillayleon.es (T.C.-S.); 2 Department of Medicine, University of Salamanca (USAL), 37007 Salamanca, Spain; 3 Institute of Biomedical Research of Salamanca (IBSAL), 37007 Salamanca, Spain; 4 Primary and Community Care, Tejares, Salamanca, 37007 Salamanca, Spain; vanehs@msn.com; 5 Pneumology Service, Zaragoza University Hospital, 50009 Zaragoza, Spain; apereztr@salud.aragon.es; 6 Primary and Community Care, Ciudad Rodrigo, 37007 Salamanca, Spain; enbarbo@gmail.com

**Keywords:** COPD, screening, forced spirometry, COPD6, PIKO6

## Abstract

**Background**: Chronic obstructive pulmonary disease (COPD) is a frequent but underdiagnosed disease, primarily due to the lack of access to forced spirometry (FS) in primary care. Portable, easy-to-use expiratory flow meters like Piko-6^®^ and COPD-6^®^ that measure FEV_1_, FEV_6_, and FEV_1_/FEV_6_ ratio provide an alternative. Given that Piko-6^®^ and COPD-6^®^ devices measure FEV_6_ but not FVC, the aim of the study is to determine the optimal cutoff value for the FEV_1_/FEV_6_ ratio of each device to avoid false negatives when these devices are used for COPD screening in primary care (PC). **Methods**: A total of 664 patients of 35 years of age or older with a cumulative tobacco consumption of 10 or more packs/year were recruited at two university hospitals. FS (gold standard) was performed and FEV_1_, FVC, and FEV_1_/FVC measurements were compared with FEV_1_, FEV_6_, and FEV_1_/FEV_6_ measurements acquired using Piko-6^®^ and COPD-6^®^ devices. The devices were compared using statistical methods including Pearson correlation coefficients, the Youden index (YI), kappa coefficient, Bland–Altman plots, and ROC curves analysis. **Results**: Correlations between FEV_1_/FEV_6_ using Piko-6^®^ and COPD-6^®^ and FEV_1_/FVC with FS were 0.79 and 0.73, respectively. Piko-6^®^ achieved the best YI in FEV_1_/FEV_6_ (0.73), whereas for COPD-6^®^, it was 0.80. Concordance between Piko-6^®^ and FS was 83.9% (kappa 0.67 ± 0.028) and for COPD-6^®^, it was 68.7% (kappa 0.42 ± 0.02). **Conclusions**: This is the first study that compares two hand-held expiratory flow meters with FS. Piko-6^®^ and COPD-6^®^ devices are effective tools for COPD detection, as their measurements provide a good correlation with FS. In order to avoid false negative results, the FEV_1_/FEV_6_ cutoff point needs to be increased to 0.73 and 0.80 with Piko-6^®^ and COPD-6^®^, respectively.

## 1. Introduction

COPD is a prevalent and progressive disease for which early diagnosis is essential. Forced spirometry (FS) is recognized as the gold standard diagnostic test; however, it is frequently underutilized or unavailable in clinical practice [1,2,3,4]. FS is only useful as long as the quality criteria are met; however, in primary care (PC), accessibility is often limited and guidelines are often not correctly followed [5,6,7]. Underdiagnosis is largely attributed to the population’s limited awareness of the disease and the low availability of FS [8,9]. According to EPISCAN (Epidemiologic Study of COPD in Spain), approximately 1,595,000 Spanish people suffer from COPD without being aware of it, which represents a serious problem [10]. FS may be technically difficult to perform. Multiple studies have shown that forced expiratory volume at six seconds of exhalation (FEV6) may serve as an appropriate substitute for forced vital capacity (FVC) [11,12,13,14,15]; execution is simpler and there is less variability because exhalatory time is reduced. This is the reason why more simple devices are accepted for evaluating FEV6 instead of FVC [16,17,18,19,20,21]. If we assume the accuracy of FEV_6_ and FEV_1_/FEV_6_ to determine obstruction, COPD screening could be performed in PC using hand-held expiratory flow meters that are easy to manipulate. Their use may help decrease underdiagnosis of the disease. The European Respiratory Society (ERS) decided in 2015 to investigate the role of these devices in the early diagnosis of COPD [22]. GesEPOC (Spanish COPD Guideline) also recommends their use in COPD screening for the at-risk patient population [23].

Significant regional disparities and methodological variations complicate the interpretation of prevalence data [24]. In Spain, 10% of adults over 40 years of age are affected by COPD [25] and this is expected to rise in the coming years. It represents the third cause of mortality in Spain and surrounding countries [26] and is responsible for a large proportion of health care costs [10,27].

The rate of underdiagnosis and undertreatment is over 70%. FS is recognized as the gold standard diagnostic test, which is set with FEV_1_/FVC < 70% post bronchodilator use (or less than the lower limit of normal) [28]. Nevertheless, it is underutilized in clinical practice both in PC and specialized care [29,30]. FVC is highly dependent on the duration of expiration, especially in elderly patients or those with airflow obstruction. Using FE has been suggested because it is more repeatable and has good concordance with FVC [13,14,15,16,17,31]. This is the reason why the National Lung Health Education Program North American Administration recommends the use of FEV_1_/FEV_6_ to detect COPD in PC clinical practice [12].

A complement to FS in the diagnostic process is hand-held expiratory flow meters, which measure FEV_1_, FEV_6_, and the FEV_1_/FEV_6_ ratio. They are inexpensive, easy to use, and do not need periodic calibrations, resulting in them being quite useful for COPD screening [4,12,32]. A meta-analysis of 11 studies showed that FEV_1_/FEV_6_ provides a sensitivity of 89% and a specificity of 98% when compared to FEV_1_/FVC. However, there is no agreement on the cutoff point for FEV_1_/FEV_6_ [13].

The proposed FEV_1_/FEV_6_ cutoff point with the COPD-6^®^ device is 0.70. According to Represas et al., the best cutoff point to diagnose obstruction is 0.75–0.80 [33]. In the studies by Sichletidis et al. [34] and Hidalgo et al. [35], the correlation between Piko-6^®^ and FS was excellent. Since FS is not always available in primary care, there is a need for research into other alternatives for analyzing lung function beyond the strict quality control in laboratories. In a recent meta-analysis, Zhou et al. [36] concluded that microrespirometers are “user-friendly, patient-friendly, inexpensive, and portable, making them suitable for primary care use and providing a feasible pathway for early diagnosis of COPD”.

The objective of our study was to assess the validity of COPD-6^®^ and Piko-6^®^ for COPD screening and to determine the best FEV_1_/FEV_6_ cutoff point for each device, i.e., the point that best allows airway obstruction to be confidently ruled out and, also, to determine the most accurate device for COPD screening. COPD screening results are important. According to the GOLD guidelines for COPD diagnosis, a diagnosis of COPD should only be made following lung function tests with FEV_1_/FVC. If obstruction is suspected using devices that measure FEV_1_/FEV_6_, the patient should be referred for appropriate spirometry. However, in the current context of limited resources in primary care, it is not always possible to perform spirometry, which is why Zhou’s recommendation is especially important. Zhou et al. [36] concluded that portable spirometers are highly accurate for diagnosing COPD.

## 2. Methods

### 2.1. Sample Size and Inclusion Criteria

The required sample size was established by the Epidat 4.2 program to be 569 patients. A total of 689 outpatients were recruited from the pneumology and tobacco cessation departments at the University Hospital of Salamanca and Clinic Hospital of Zaragoza (Spain) between March 2015 and January 2017. All patients participating in the study signed an informed consent form approved by the clinical research ethics committee. The inclusion criteria encompassed subjects aged over 35, smokers or ex-smokers of over 10 packs/year, with or without respiratory symptoms. The exclusion criteria were the inability to undertake valid and repeatable spirometry measurements (quality A, B, and C) and presenting any kind of medical condition or absolute contraindications to perform the test.

### 2.2. Development of Study Procedures

All spirometry tests were conducted following the ERS/ATS standards for spirometry 2005 by qualified operators at lung function laboratories (trained doctors, nurses, or physician assistants). The variables collected included: anthropometric data, packs/year, symptoms, FEV_1_, FVC, and FEV_1_/FVC ratio obtained via FS (Jaeger 920 Masterlab, Würzburg, Germany) measured in milliliters and in percentage with respect to the reference values for FEV_1_, FEV_6_, and FEV1/FEV6 ratio obtained by the hand-held expiratory flow meters Vitalograph COPD-6^®^ and Piko-6^®^. In addition, to evaluate the subjective difficulty of performing the three tests (FS, COPD-6^®^, and Piko-6^®^), the results were measured on a visual score from 0 to 10. All subjects followed the same test sequence: (1) FS, administration of Albuterol 400 mcg through a spacer chamber and 15 min later BDT; (2) a test with COPD-6^®^; and (3) a test with Piko-6^®^. Once completed, participants were required to evaluate the subjective difficulty of the tests using a visual score from 0 to 10. The staff who administered the tests ensured that patients understood and correctly performed the procedure for each test and rested adequately between each test.

### 2.3. Statistical Analysis

Different statistical tests were applied depending on the specific variable analyzed. A significance level of 0.05 was applied across all analyses. The statistical analyses used to compare the devices were Pearson correlations, Youden index (YI), kappa coefficient, Bland–Altman plots, and ROC curves, and the analyses were conducted using IBM SPSS 23 version software.

## 3. Results

### Sample Studied and Main Results

A total number of 664 patients from the total of 689 recruited subjects met the criteria to be taken into account: 491 males (74%) and 173 females (26%). The average age was 61.5 ± 11 years; 338 are ex-smokers (51%) and 326 are current smokers (49%); and the average was 41.2 ± 22 packs/year of smoking (range 10–150). Table 1 shows individuals classified as healthy or ill according to both hand-held expiratory flow meters and FS. The alternative devices classified more healthy patients than FS by using FEV_1_/FEV_6_ 0.7. FS detected 411 patients with COPD (62%), Piko-6^®^ detected 340 (51%) and COPD-6^®^ detected 209 (31.5%).

Table 2 includes measurements in percentages and milliliters for each variable. We observed that absolute measurements and percentages for FEV_1_ and FEV_1_/FEV_6_ recorded by the portable expiratory flow meters were lower. A contingency table with Piko-6^®^, COPD-6^®^, and FS was created. Obstruction was defined as FEV1/FVC < 70%. Piko-6^®^ showed a sensitivity of 78.35% and a specificity of 93%, with a positive predictive value of 94.7% and a negative predictive value of 72.5%. The YI was 0.71. Moreover, COPD-6^®^ obtained a sensitivity of 50.12%, a specificity of 98.8%, a positive predictive value of 98.5%, a negative predictive value of 54.9%, and a YI of 0.49. Intra-cluster correlation was used to analyze concordance between the parameters, as shown in Table 3 (adapted from Hernández-Mezquita et al. [37]).

**Figure 1:**
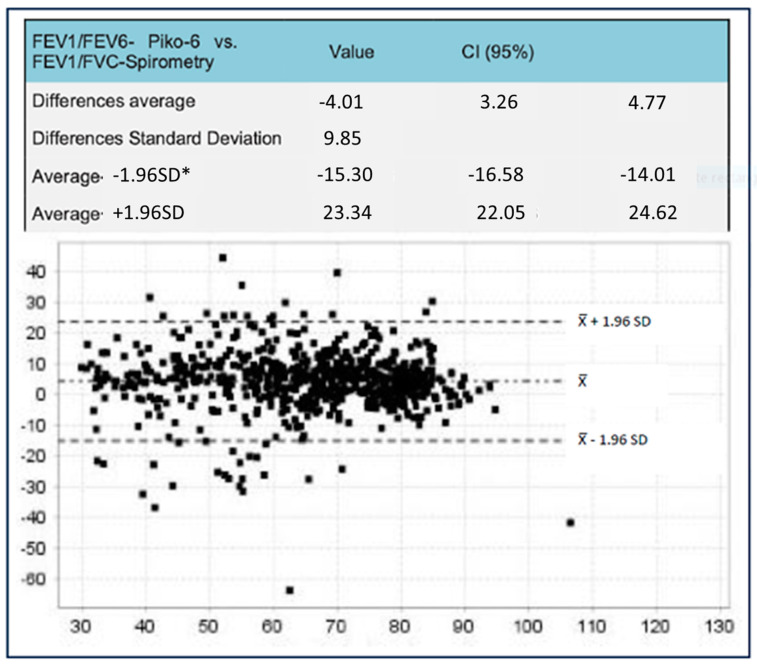
Bland-Altman plots of the mean differences between FEV_1_/FEV_6_ obtained by Piko-6 vs. FEV_1_/FVC via FS. * Standard Deviation.

**Figure 2:**
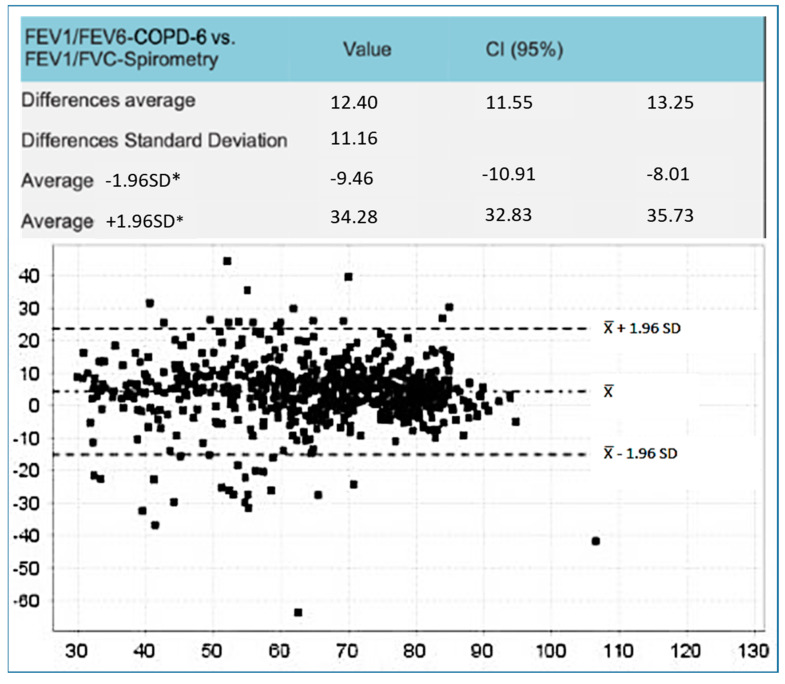
Bland–Altman plots of the differences between FEV1/FEV6 obtained by COPD-6 vs. FEV1/FVC via FS. * Standard Deviation.

The Bland–Altman plots represent the differences observed (Figure 1 and Figure 2). The difference between FEV_1_/FEV_6_ and FEV_1_/FVC was smaller with Piko-6^®^. The biggest differences were observed between FEV6 by using both devices and FVC with FS (4.01; CI 95% 3.26–4.7 vs. 12.4; IC 95% 11.5–13.25).

**Figure 3:**
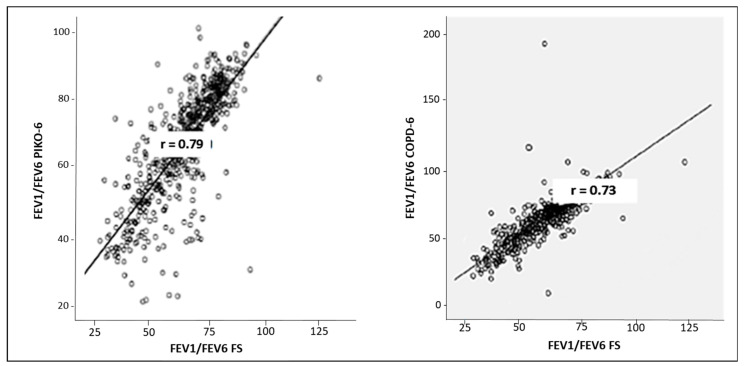
Correlation between FEV1/FEV6 obtained by Piko-6 (**left**) and COPD-6 (**right**) and FEV1/FVC obtained via FS.

**Figure 4:**
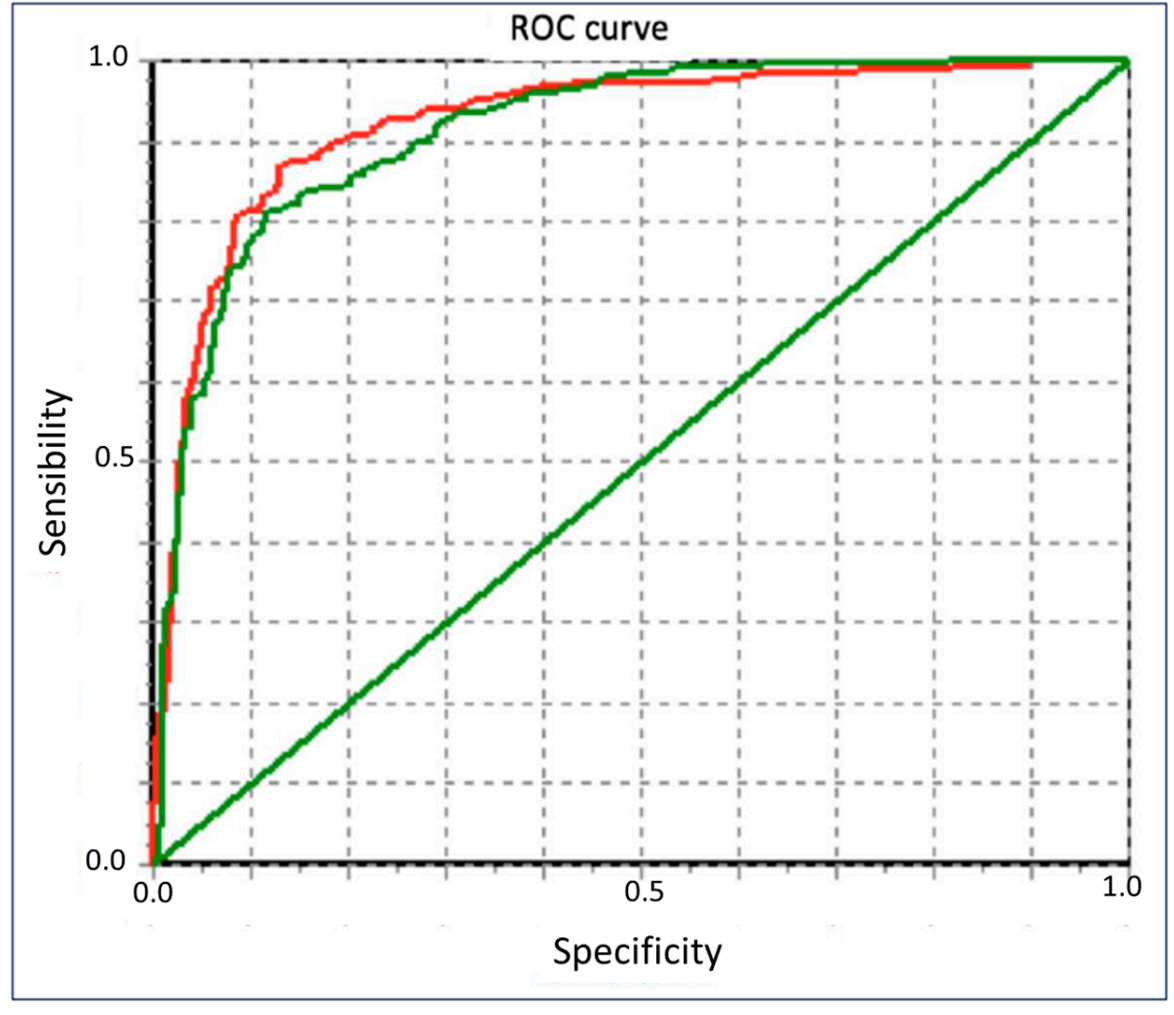
Comparison of FEV1/FEV6 ROC curves obtained by Piko-6 (red, test 1) and COPD-6 (green, test 2).

The Pearson correlation index of FEV_1_ between FS and Piko-6^®^ and between FS and COPD-6^®^ was 0.94 and 0.97, respectively (Table 4). Correlations between FEV_1_/FEV_6_ by using Piko-6^®^ and COPD-6^®^ were 0.79 and 0.73, respectively (Figure 3). The ROC curves graphically show the FEV_1_/FEV_6_ sensitivity and specificity of Piko-6^®^ and COPD-6^®^, using FEV_1_/FVC < 70% as a reference. The area under the curve (UAC) was 0.922 for Piko-6^®^ and 0.913 for COPD-6^®^ (Chi-squared test *p* = 0.438) (Figure 4).

Table 5 shows the sensitivity, specificity, NPV, PPV, and YI for both devices at the different cutoff points of the FEV_1_/FEV_6_ ratio. The YI of Piko-6^®^ (greater relation between sensitivity and specificity) was better at the cutoff of <73% (0.74). When using COPD-6^®^, the sensitivity value was lower, so the cutoff point was 80% (the cutoff point of the FEV1/FEV6 ratio at which a better Youden index is obtained with each device has been marked in bold). The concordance observed between Piko-6^®^ and FS was 83.9%, with a kappa value of 0.67 ± 0.028. Moreover, COPD-6^®^‘s concordance was 68.7% and the kappa value was 0.42 ± 0.02. With regard to the visual scale of subjective difficulty of the tests, Piko-6^®^ showed an average of 2.55, COPD-6^®^ showed an average of 2.78, and FS showed an average of 3.87. More than 75% of patients reported difficulty ≤ 3 and less than 2% ≥ 7 with Piko-6^®^. With COPD-6^®^, more than 30% of patients showed a difficulty level ≤ 3, and with FS, this was less than 50%. Almost 10% of patients reported a ≥ 7 level of difficulty.

## 4. Discussion

COPD is underdiagnosed, so screening tools are needed to improve disease management. FS undertaken in PC is often unreliable due to the challenges of managing the procedure. Artificial intelligence can help improve outcomes in the diagnosis and management of COPD by facilitating the analysis of spirometry data, optimizing early detection, and reducing errors in the evaluation process in primary care [38]. The European Respiratory Society (ERS) has proposed investigating the utility of hand-held expiratory flow meters as an alternative [22]. Several studies have examined the utility of FEV_1_/FEV_6_ as an alternative to FEV_1_/FVC [13,32,39]. Recently, our research team also published a scientific paper [36] about the accuracy of PIKO-6^®^ and COPD-6^®^ devices in COPD screening, complementary to this article, that focuses on the analysis of cutoff points to avoid false negatives with both devices.

This research was conducted to assess the validity of Piko-6^®^ and COPD-6^®^ in the diagnosis of airway obstruction and to identify which is the most reliable. To achieve this aim, FEV_1_, FEV_6_, and FEV_1_/FEV_6_ measurements obtained by Piko-6^®^ and COPD-6^®^ were compared with FEV_1_, FVC, and FEV_1_/FVC obtained via FS.

Jing et al.’s [13] meta-analyses established that FEV_1_/FEV_6_ has a sensitivity of 89% (IC 95%: 83–93%) and a specificity of 98% (IC 95%: 95–99%) in relation to FEV_1_/FVC. The FEV_1_/FEV_6_ AUC ROC to detect airway obstruction was excellent at 0.97 and the authors concluded that FEV_1_/FEV_6_ could substitute FEV_1_/FVC. Frith et al. [32], Van den Bemt et al. [38], and Kauffman et al. [40] showed good results with Piko-6^®^, although they used different methods and targets in their studies. There are two studies with similar objectives and designs to our project: Hidalgo et al. [35], who studied Piko-6^®^, and Represas et al. [33], who studied COPD-6^®^. In our study, FEV_1_ and FEV_6_ obtained by COPD-6^®^ and Piko-6^®^, both in milliliters and percentages were lower than FEV1 and FVC collected via FS (*p* < 0.001). Our COPD-6^®^ results did not substantially differ from those of Represas et al. [33]. In both studies, FEV_1_/FEV_6_ was higher than FEV_1_/FVC (*p* < 0.001), which was expected because FEV_6_ was lower than FVC. COPD-6^®^ FEV_1_ measurement was also lower than that obtained via FS. Based on this, the cutoff point for diagnosing obstruction should be reconsidered from 0.7, since COPD-6^®^ did not detect obstruction in almost half of patients. In our study, we observed less dispersion of FEV_1_ with respect to FS than Represas, an average difference of 144 mL (IC 95%: 126–162) vs. 167 mL (IC 95%: 144–190). In contrast, in relation to the FEV_6_ value, we found slightly greater dispersion. Represas observed a good correlation between FEV_1_, FEV_6_, and FS, which was similar to our study, and a higher correlation between FEV_1_/FEV_6_ and FS. In summary, we observed a good correlation between COPD-6^®^ and FS, particularly with FEV_1_ (r = 0.97) measurement, with consistent results similar to those published by Represas [33].

In both Hidalgo’s study and our present project in relation to Piko-6^®^, FEV1 and FEV6 values were also lower than FEV_1_ and FVC determined via FS (*p* < 0.001); however, no significant differences were observed between FEV1/FEV6 and FEV1/FVC. When we analyzed the FEV1 dispersion of Piko-6^®^ versus FS by using Bland–Altman plots, the average dispersion (8%; IC 95%: 5–12) was higher than in Hidalgo et al.’s study (4%; IC 95%: 2–6) [35]. The average dispersion of the FEV1/FEV6 ratio was 4% (IC 95%: 3–4) and in Hidalgo et al.’s study, it was 0.7% (IC 95%: 0.1–1.5). Hidalgo et al. [35] reported a good correlation between FEV_1_, FEV_6_, and FEV_1_/FEV_6_. In contrast, we observed the best correlation with FEV1 (r = 0.94 versus r = 0.87) and slightly worse with the ratio (r = 0.79 versus 0.94) [35]. This correlation could be considered excellent based on linear regression lines. We tried to find out the best FEV_1_/FEV_6_ cutoff point in terms of sensitivity and specificity to detect obstruction (defined as FEV1/FVC < 0.7 post bronchodilator test according to clinical guidelines). However, the FEV_1_/FEV_6_ acquired by the hand-held expiratory flow meter was greater than FEV_1_/FVC. This is the reason why a higher cutoff should be considered. To achieve this aim, we used the YI. The cutoff point in the studies by Represas et al. [33], Fritz et al. [32], Hidalgo et al. [35], and Van de Bemt et al. [38] varied between 0.70 (Hidalgo) and 0.78 (Represas). In their research, Fritz and Van de Bemt obtained intermediate values of 0.75 and 0.73, respectively. We noticed different cutoff points given that the sensitivity and specificity of each device were different. The Piko-6^®^ cutoff point with the best YI was 0.73, and with COPD-6^®^, it was 0.8 and had considerably lower sensitivity. Represas et al. [33] concluded that when using COPD-6^®^, a cutoff point of 0.7 was not valid for COPD screening and that a cutoff point of 0.75–0.80 was needed, which was in accordance with our findings. Nevertheless, Hidalgo et al. [35] observed that FEV_1_/FEV_6_ with the best YI was 0.70 when using Piko-6^®^, which was slightly inferior to our YI of 0.73. Nonetheless, this is not a limitation for using this device for COPD screening. To sum up, the best YI to FEV_1_/FEV_6_ ratios were 0.73 for Piko-6^®^ and 0.80 for COPD-6^®^, similar to the results of other authors. ROC curves were performed to compare FEV_1_ and FEV_1_FEV_6_. FEV_1_/FVC < 70% was used as reference. We noticed a UAC of 0.91 for FEV_1_/FEV_6_ with COPD-6^®^, slightly inferior to that in Represas’ study (UAC of 0.97) [33]. With regard to Piko-6^®^, the UAC (0.84 for FEV_1_ and 0.92 for FEV_1_/FEV_6_) and UAC of Hidalgo [35] (0.8 and 0.92, respectively) showed an excellent correlation with FS.

When analyzing concordance with the kappa index between both devices and FS, the results were better with Piko-6^®^ than with COPD-6^®^ (0.55 and 0.43, respectively). These results were slightly worse than those obtained by Hidalgo with Piko-6^®^ [35]. However, both devices showed an odds ratio (OR) higher than 20, which is the minimum needed to validate a test. Piko-6^®^ showed a concordance of 83.9% to classify individuals as ill (positive diagnosis of COPD) or healthy (COPD diagnosis excluded), with a kappa value of 0.67 ± 0.028 and 68.7% and kappa of 0.42 ± 0.02 with COPD-6^®^. Therefore, Piko-6^®^ has good concordance and COPD-6^®^ has moderate concordance. Consequently, with a cutoff of FEV_1_/FEV_6_ < 0.70, Piko-6^®^ did not detect a small percentage of patients that FS diagnosed as having COPD, a finding similar to that observed by Fatima et al. [41] in 2017. This percentage was superior with COPD-6^®^, which necessitates increasing the FEV_1_/FEV_6_. In clinical practice, it is important to bear in mind the subjective difficulty that patients observe when performing the tests. Only Hidalgo et al. [35] have evaluated this aspect. The subjective difficulty average was 2.55 points with Piko-6^®^, 2.78 with COPD-6^®^, and 3.87 with FS. These results were significant and concordant with those of Hidalgo et al. [35]. We could state that patients preferred performing tests using less difficult hand-held expiratory flow meters than using FS. The reasons could be the greater simplicity of the device, the fewer maneuvers needed, or the decreased time for the exhalatory maneuver. This study provides real-world evidence to identify optimal practices for COPD diagnostic practices; such practices ensure that physicians have convenient access to up-to-date evidence when they encounter a symptomatic patient likely to have COPD. Chen et al. [42] concluded in their study published in 2021 that microspirometry was accurate and had higher clinical utility.

In conclusion, the effectiveness of hand-held expiratory flow meters for COPD screening seems to be clear given the accuracy of the measurements and their acceptance by patients in helping to reduce underdiagnoses of the disease and alleviating workloads in pulmonary function laboratories. This is the first study that compares two hand-held expiratory flow meters with FS. Although FEV_1_ and FEV_6_ measurements obtained with hand-held expiratory flow meters were lower than FEV_1_ and FVC values measured with FS, Piko-6^®^ and COPD-6^®^ devices are useful for COPD screening because correlation with their FS is good. An FEV_1_/FEV_6_ cutoff point of 0.7 was obtained by the hand-held expiratory flow meters as COPD screening had false negative results, so it should be increased: 0.73 for Piko-6^®^ and 0.80 for COPD-6^®^.

The quality and accuracy of performing and interpreting spirometry measurements in primary care is variable; therefore, microspirometers are currently useful. Perhaps, in the near future, artificial intelligence (AI) could enhance the performance of primary care physicians in spirometry interpretation [43], although the difficulty for patients in performing the test will remain an issue.

## Figures and Tables

**Table 1:** Patients classified as having COPD based on GOLD criteria using the three devices: forced Spirometry (FS), Piko-6^®^, and COPD-6^®^.

		Frequency	Percentage	Valid Percentage	Accumulated Percentage
**Forced Spirometry**					
Valid	ILL (COPD)	411	61.9	61.9	61.9
	HEALTHY	253	38.1	38.1	100.0
	Total	664	100.0	100.0	
**Piko-6^®^**					
Valid	ILL (COPD)	340	51.2	51.2	51.2
	HEALTHY	324	48.8	48.8	100.0
	Total	664	100.0	100.0	
**COPD-6^®^**					
Valid	ILL (COPD)	209	31.5	31.5	31.5
	HEALTHY	455	68.5	68.5	100.0
	Total	664	100.0	100.0	

**Table 2:** Absolute measurements and percentage of FEV_1_, FVC, and FEV_6_ obtained through forced spirometry (FS) and by the Piko-6^®^ and COPD-6^®^ devices (n = 664 subjects).

	Average	Standard Deviation
FEV_1_ (%) by Piko-6^®^	71.06	26.83
FEV_1_ (%) by COPD-6^®^	79.94	30.04
FEV_1_ (%) by FS	83.58	46.53
FEV_6_ (%) by Piko-6^®^	81.15	48.52
FEV_6_ (%) by COPD-6^®^	82.52	24.21
FVC (%) by FS	100.08	106.51
FEV_1_ (cc) by Piko-6^®^	2000.87	886.98
FEV_1_ (cc) by COPD-6^®^	2158.71	969.35
FEV_1_ (cc) by FS	2303.30	987.97
FEV_6_ (cc) by Piko-6^®^	2936.43	998.72
FEV_6_ (cc) by COPD-6^®^	2800.87	1025.12
FVC (cc) by FS	3577.03	1042.26

**Table 3:** Concordance and correlation coefficient between simple hand-held expiratory flow meters and FS, adapted from Hernández-Mezquita et al. [37].

Piko-6^®^ vs. Spirometry	Correlation	CI (95%)	
FEV_1_, cc	0.8916	0.8748	0.9062
FEV6, cc (FVC, cc)	0.6456	0.599	0.6879
FEV_1_, %	0.3756	0.3084	0.439
FEV6, % (FVC, %)	0.0133	0	0.0892
FEV_1_/FVC	0.7663	0.733	0.7959
**COPD-6^®^ vs. Spirometry**			
FEV_1_, cc	0.9599	0.9535	0.9655
FEV6, cc (FVC, cc)	0.6743	0.6306	0.7137
FEV_1_, %	0.4321	0.3682	0.492
FEV6, % (FVC, %)	0.0526	0	0.1281
FEV_1_/FVC	0.4936	0.4339	0.549

**Table 4:** Pearson correlation between COPD-6^®^, Piko-6^®^, and spirometry measurements.

PEARSON CORRELATION BETWEEN COPD-6®, PIKO-6®, AND FS		
Piko-6^®^ FEV_1_ (cc)	Pearson Correlation	0.946
	Sig. (bilateral)	0.000
COPD-6^®^ FEV_1_ (cc)	Pearson Correlation	0.971
	Sig. (bilateral)	0.000
Piko-6^®^ FEV_1_/FEV_6_	Pearson Correlation	0.796
	Sig. (bilateral)	0.000
COPD-6^®^ FEV_1_/FEV_6_	Pearson Correlation	0.737
	Sig. (bilateral)	0.000

**Table 5:** Sensibility (S), specificity (E), positive predictive value (PPV), negative predictive value (NPV), and Youden index (YI) of Piko-6^®^ and COPD-6^®^.

FEV_1_/FEV_6_ Piko-6^®^ (%)	YI	S (%)	E (%)	PPV (%)	NPV− (%)
<68	0.63	72.99	90.32	91.23	70.79
<69	0.64	73.7	90.42	92.24	69.01
<70	0.71	78.35	92.89	94.71	72.53
<71	0.69	75.63	92.89	95.4	66.14
<72	0.73	77.29	96.12	97.79	65.56
<73	0.74	78.21	95.41	97.6	64.71
<74	0.72	79.34	92.78	96.73	62.55
<75	0.74	81.91	92.44	96.88	64.11
<76	0.72	83.04	88.54	95.9	61.78
<77	0.74	85.06	88.73	96.52	61.76
<78	0.69	84.87	84.43	96.03	55.68
<79	0.68	84.55	83.17	96.55	49.12
<80	0.63	85.52	77.38	96.31	43.62
**FEV_1_/FEV_6_** **COPD-6^®^ (%)**					
<68	0.44	46.49	97.85	96.76	56.99
<69	0.44	47.15	96.93	95.96	54.29
<70	0.46	47.78	97.89	97.61	50.99
<71	0.46	48.52	97.78	97.71	49.33
<72	0.49	50.22	99.03	99.14	47.22
<73	0.5	51.5	98.47	98.77	45.95
<74	0.5	52.69	97.78	98.46	43.46
<75	0.55	56.3	98.26	98.93	44.01
<76	0.56	57.79	98.09	98.99	41.85
<77	0.57	58.81	98.59	99.35	39.44
<78	0.58	59.23	98.36	99.38	35.19
<79	0.58	61.46	97.03	99.14	31.11
<80	0.59	64.14	95.24	98.94	27.78

## Data Availability

No restrictions on data availability. Data statement: The data are available at https://scholar.archive.org/work/ng6ecpnosrftzpkzvkyjhjrhtu (accessed on 21 December 2024).
